# Umbelliprenin via increase in the *MECP2* and attenuation of oxidative stress mitigates the autistic-like behaviors in mouse model of maternal separation stress

**DOI:** 10.3389/fphar.2023.1300310

**Published:** 2024-01-08

**Authors:** Parnian Karimi, Mehryar Shahgholian Ghahfarroki, Zahra Lorigooini, Mehrdad Shahrani, Hossein Amini-Khoei

**Affiliations:** Medical Plants Research Center, Basic Health Sciences Institute, Shahrekord University of Medical Sciences, Shahrekord, Iran

**Keywords:** maternal separation stress, Autism spectrum disorder, umbelliprenin, MeCP2, oxidative stress

## Abstract

**Introduction:** Autism spectrum disorder (ASD) is a complex neurodevelopmental condition. Maternal separation (MS) stress is an early-life stress factor associated with behaviors resembling Autism. Both *MECP2* and oxidative stress are implicated in the pathophysiology of Autism. Umbelliprenin (UMB) is a coumarin compound with various pharmacological properties. Our study aimed to investigate the potential effects of UMB in mitigating autistic-like behaviors in a mouse model subjected to MS stress, focusing on probable alterations in *MECP2* gene expression in the hippocampus.

**Methods:** MS paradigm was performed, and mice were treated with saline or UMB. Behavioral tests consisting of the three-chamber test (evaluating social interaction), shuttle box (assessing passive avoidance memory), elevated plus-maze (measuring anxiety-like behaviors), and marble-burying test (evaluating repetitive behaviors) were conducted. Gene expression of *MECP2* and measurements of total antioxidant capacity (TAC), nitrite level, and malondialdehyde (MDA) level were assessed in the hippocampus.

**Results:** The findings demonstrated that MS-induced behaviors resembling Autism, accompanied by decreased *MECP2* gene expression, elevated nitrite, MDA levels, and reduced TAC in the hippocampus. UMB mitigated these autistic-like behaviors induced by MS and attenuated the adverse effects of MS on oxidative stress and *MECP2* gene expression in the hippocampus.

**Conclusion:** In conclusion, UMB likely attenuated autistic-like behaviors caused by MS stress, probably, through the reduction of oxidative stress and an increase in *MECP2* gene expression.

## Introduction

Autism spectrum disorder (ASD) encompasses a diverse range of neurodevelopmental disorders characterized by challenges in social communication and interaction, as well as repetitive and restricted patterns of behavior and interests (Wen et al., 2017). Various factors such as epigenetic, metabolic, and environmental conditions are ASD pathogenic factors (Faras et al., 2010). Previous studies have determined that neonatal exposure to psychosocial difficulties, such as maternal separation (MS) stress, significantly disrupts brain development, consequently increasing susceptibility to neuropsychiatric disorders (Amiri et al., 2016; Amini-Khoei et al., 2019). Ample evidence has been demonstrated that MS could lead to neurotransmission, neuroendocrine, and neurostructural alterations in the central nervous system (CNS) (Gildawie et al., 2021). It has been well-determined that MS via neurotransmission and neurostructural modifications in the brain provoked autistic-like behaviors in rodents, indicating that the MS paradigm could be considered as an experimental model of Autism in rodents (Mansouri et al., 2021; Farzan et al., 2023; Khaledi et al., 2023; Moghaddam et al., 2023; Yadollahi-Farsani et al., 2023). MS, with its abovementioned changes in the hippocampus, causes mood-behavioral disorders (Arabi et al., 2021). Studies on autistic patients represented irregular hippocampal patterns (Xu et al., 2020). Studies on the experimental models have determined hippocampal alterations in Autism, indicating the pivotal role of the hippocampus in the pathophysiology of ASD (Zhong et al., 2020; Lee et al., 2023). Methyl-CpG-Binding Protein 2 (*MECP2*) regulates neurons that modulate social interactions (Pejhan and Rastegar, 2021). Some cases of neurodevelopmental disorders like Autism and Rett syndrome may be linked to mutations or dysregulation of the *MECP2* gene (Zoghbi, 2005; Calfa et al., 2011), suggesting that *MECP2* is involved in the pathophysiology of Autism (Neul, 2022). *MECP2* regulates neurophysiological functions implicated in developing neurons and synaptic plasticity (Sánchez-Lafuente et al., 2022). Specifically, researchers have observed that methylation at a specific region of the *MECP2* gene, which causes a decrease in the level of *MECP2* protein in the brain, is associated with Autism (Neul, 2022). Preclinical studies have determined that hippocampal *MECP2* knockdown provoked autistic-like behaviors in rats (Choi et al., 2022). Thus, *MECP2* could consider as a new target to introduce new agents as well as explore the efficacy of therapeutics in ASD.

Coumarins are natural benzopyrene derivatives abundantly present in various plant sources (Iranshahi et al., 2009; Shakeri et al., 2014). They possess a wide range of pharmacological activities.

Umbelliprenin (UMB) is a sesquiterpene Coumarin compound found in Ferula species. Extensive research has demonstrated that UMB possesses various pharmacological properties (Hashemzaei et al., 2020). Numerous studies have confirmed its diverse effects, including antioxidative, neuroprotective, proapoptotic, and anti-inflammatory properties (Hashemzaei et al., 2015). Researchers have investigated its impact on neuropathic pain and found that it effectively mitigates oxidative stress while providing neuroprotection (Shahraki et al., 2020). Additionally, UMB has shown anti-inflammatory properties and acts as a modulator of cytokine secretion (Khaghanzadeh et al., 2017).

In this study, we aimed to investigate the potential of UMB to mitigate autistic-like behaviors in a mouse model of MS stress, with a specific focus on assessing its effect on the alterations of *MECP2* gene expression in the hippocampus.

## Materials and methods

### Animals and maternal separation paradigm

Pregnant Naval Medical Research Institute (NMRI) mice were obtained from the Institute Pasteur in Iran. The animals were housed in laboratory conditions with standard parameters, including a 12-hour light/dark cycle (with lights turned on at 8:00 a.m.), a consistent temperature of 21°C ± 2 °C, and free access to food and water. The birthday (pups with an average weight of about 3 g) was designated as postnatal day (PND) 0. From PND2, the neonates were separated from their mothers for 3 h each day (from 10 a.m. to 1 p.m.) until PND14. Subsequently, the neonates were returned to their mothers’ cages until PND25 (Lorigooini et al., 2020a). To avoid the litter effect, pups of each mouse were accidentally numbered on PND 25 and were randomly assigned to the experimental groups. Forty-five maternally separated male mice were randomly divided into three groups (n = 15). Additionally, fifteen NMRI male mice not exposed to the MS model were selected as the control group. By establishing a bilateral alpha of 0.05 and a confidence interval of 90%, 15 mice were considered for each experimental group based on a sample size calculation formula (Chen et al., 2022).

### Study design

All agents were administered via the intraperitoneal (i.p.) route for seven consecutive days from PND 51–53 until PND 58–60. The behavioral experiments related to autistic-like behaviors were conducted immediately after the treatments were completed and carried out between 09:00 a.m. and 05:00 p.m. (light phase). Finally, the mice were sacrificed under deep anesthesia using diethyl ether, and their hippocampi were dissected for molecular analysis (Lorigooini et al., 2021). The dose and administration time were selected based on previous studies and our pilot study (Rashidi et al., 2018; Shahraki et al., 2020).

The control group (Group 1) received normal saline (1 mL/kg). The second to fourth groups consisted of the MS mice treated with normal saline (1 mL/kg), UMB (12.5 mg/kg), and UMB (25 mg/kg), respectively. It has been established that animals display low subject-to-subject variation (Jiang et al., 2017). To minimize animal suffering and diminish the number of mice used, based on the formula introduced by Charan et al., five mice in each group is the satisfactory limit and, therefore, can be considered an adequate sample size to see the effect of the drug in animal studies (Charan and Kantharia, 2013). Since behavioral tests impose different levels of stress on animals, to minimize the impact of stress reactivity, from 15 mice in each experimental group, five mice were subjected to the three-chamber sociability test, five mice were subjected to the shuttle box test, and five mice subjected to the EPM and MBT (7).

### Behavioral tests

#### Evaluation of sociability and social preference indexes

The three-chamber test was employed to assess social behaviors. A plexiglass box was divided into three chambers: a central section and two side chambers. During the habituation, sociability, and social preference phases, mice freely explored the box. In the habituation phase, mice were placed in the central chamber for 5 min to acclimatize to the environment. Two cylindrical wire cages were placed in the two side chambers for the sociability phase. In the next step, one wire cage contained a stranger of the same sex and age mouse (novel mouse 1 or social stimulus 1), and the time spent exploring each chamber was measured for 10 min. The other wire cage remained empty. The time spent directly interacting with the social stimulus and the empty chamber (non-social stimulus) was recorded. The sociability index (SI) was calculated as (social stimulus 1 - non-social stimulus)/(social stimulus 1 + non-social stimulus). For the social preference index (SPI), another unfamiliar mouse (new mouse 2 or social stimulus 2) was placed in a different empty wire cage, and the time spent in each chamber was recorded for 10 min. The SPI was determined using the following formula: (social stimulus 2 - social stimulus 1)/(social stimulus 2 + social stimulus 1) (Amini et al., 2023; Pang et al., 2023).

#### Evaluation of passive avoidance memory

The shuttle box is a device used to assess passive avoidance memory. The mice were placed in the box, and adaptation of the mice was completed over the first 2 days. Mice were allowed to explore the apparatus for 5 min. On the third day, the mice were placed in the bright chamber, and after 2 min, the door was opened. The door was closed when the mice entered the dark section, and an electrical shock (1 mA/second) was administered. The dark chamber latency was recorded as the initial latency (T1). The same procedure was repeated on the fourth day without the electrical shock, and the second latency was recorded (T2) (Farzan et al., 2023).

#### Evaluation of repetitive behaviors

The Marble Burying Test (MBT) assessed repetitive behaviors in rodents. For the test, each mouse was placed in a clean cage containing 20 marbles arranged in a uniform grid pattern on fresh, unscented mouse bedding material with a depth of 5 cm and allowed to explore for 20 min. The number of marbles buried (covered with at least two-thirds of the bedding) was then calculated and recorded for each mouse. To avoid any potential bias, the test cage and marbles were carefully cleaned with 70% ethanol between each test, and fresh bedding material was used for each test (Farzan et al., 2023).

#### Evaluation of anxiety-like behavior

The Elevated Plus Maze (EPM) is an appropriate test for assessment of rodents’ anxiety-like behavior. The maze was constructed from black opaque Plexiglas and consisted of two open arms (30 × 5 cm) and two closed arms (30 × 5 × 15 cm), which were connected by a central platform (5 × 5 cm). During the test, each mouse was individually placed in the center of the maze, facing one of the closed arms, for 5 min, allowing it to explore the maze. The time spent in each arm, including the open and closed arms, and the number of entries into each arm were recorded. An arm entry was defined as placing all four paws into an arm (Sadeghi et al., 2023). In addition, the anxiety index was calculated as follows: Anxiety Index = 1 − ([Open arm time/Test duration] + [Open arms entries/Total number of entries]/2) (Moreno-Martínez et al., 2022). After each trial, the maze was cleaned with 70% ethanol to eliminate any odor cues left by the mice. The test was conducted in a quiet environment with controlled lighting conditions to minimize external disturbances.

#### Nitrite assay

Initially, the mice were anesthetized with diethyl ether and sacrificed, and the hippocampus was removed and immediately placed into liquid nitrogen. The Griess reaction method was used to assess the nitrite level. Hippocampal homogenates were prepared, and nitrite concentrations were determined using a colorimetric assay based on the Griess reaction. In brief, 100 μL of samples were loaded into each well and mixed with 100 μL of Griess reagent. After 10 minutes of incubation at room temperature, the automated plate reader measured the absorbance at 540 nm. The nitrite level was determined using a standard curve of sodium nitrite (Sigma, United States of America) and reported as micromole per mg protein (Wopara et al., 2021).

#### Measurement of malondialdehyde (MDA) level

The MDA level in the hippocampus was measured using the previously described method. To do this, 100 μL of hippocampus supernatant aliquots were mixed with 900 μLs of Tris-KCl buffer, and then 500 μL of 30% TCA was added. Afterward, 500 μL of thiobarbituric acid (TBA) (0.75%) was added and permitted to be heated in a water bath at 80 C for 45 min. The mixture was centrifuged (3,000 rpm g, 5 min), and the supernatant’s absorbance was read at 562 nm using an ELISA reader. The MDA level is reported as a nanomole of MDA per mg protein (Nagababu et al., 2010).

#### Measurement of total antioxidant capacity (TAC)

The ferric-reducing ability of plasma (FRAP) method was used to determine the TAC in the hippocampus. This method measured TAC in the hippocampal samples using the previously reported method at 37°C and pH 3.6. After 30 min, absorbance was measured and registered as a percentage of the combined ferric reducing/antioxidant potency of the antioxidants in protein, with the findings given as micromol Fe2+/mg protein (Benzie and Strain, 1999; Nasiri-Boroujeni et al., 2021).

#### Quantitative Real-Time polymerase chain reaction (qRT-PCR)

The gene expression of *MECP2* in the hippocampus was measured using Real-Time PCR. After collecting the hippocampal tissue, total RNA was extracted using RNX-plus. The RNA was then reverse-transcribed into cDNA using a PrimeScript RT reagent kit (Takara Bio, Inc., Otsu, Japan). Gene-specific primers and a fluorescent probe for *MECP2* were designed and optimized. Real-time PCR was done on the cDNA samples using a light cycler instrument (Roche Diagnostics, Mannheim, Germany) (Takara Bio). The results were analyzed using the 2^−ΔΔCT^ method to calculate the relative gene expression levels of *MECP2* in the hippocampus. The housekeeping gene B2M was used as a reference gene to normalize the gene expression levels (Omidi-Ardali et al., 2019; Lorigooini et al., 2021). Table 1 presents primer sequences.

**TABLE 1:** primer sequences.

Gene	Forward	Reverse
*MECP2*	TGT​ATG​ATG​ACC​CCA​CCT​TGC​C	TCC​CTC​TCC​CAG​TTA​CCG​TGA​A
B2m	CGT​GAT​CTT​TCT​GGT​GCT​TGT​C	GGA​AGT​TGG​GCT​TCC​CAT​TCT

#### Data analysis

Kolmogorov–Smirnov test was applied to evaluate the normal distribution of data, resulting in parametric data. Brown-Forsythe test was used for the evaluation of data homogeneity. Data were expressed as mean ± S.E.M and analyzed with one-way variance analysis (ANOVA) followed by Tukey’s *post hoc* test. Results were deemed statistically significant at *p* < 0.05.

## Results

### Effects of UMB on passive avoidance memory in the shuttle box test

**FIGURE 1:**
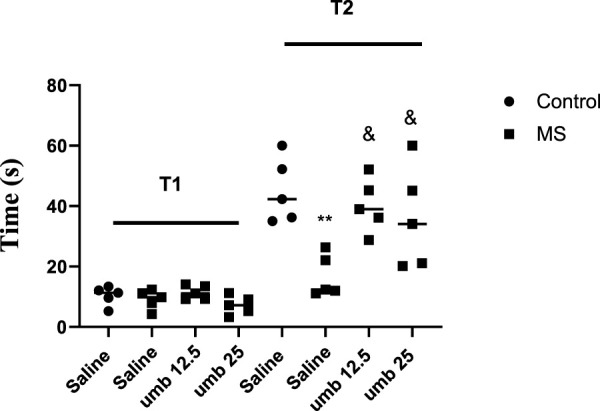
The effect of UMB on the initial and step-through latencies in the passive avoidance response in the shuttle box test. Values were calculated for a sample of 5 mice and reported as the mean ± S.E.M. The statistical analysis employed a one-way ANOVA followed by Tukey’s post-test. ^**^ *p* < 0.001 in compared to the control group, ^&^ *p* < 0.05 in compared to the saline-treated MS group.

The results indicated no notable difference among the experimental groups in the initial phase (T1) of the shuttle box test. However, in the second phase of the test (T2), the results showed a marked reduction in the MS group compared to the control group (*p* < 0.01). We observed that UMB at doses of 12.5 and 25 mg/kg significantly increased T2 compared to the saline-treated MS group (*p* < 0.05) (Figure 1).

### Effects of UMB on repetitive behavior in the marble burying test

**FIGURE 2:**
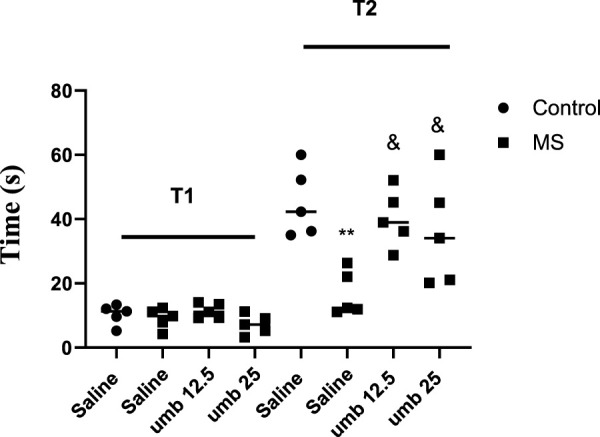
The effect of UMB on the number of marbles buried. The values were calculated for a sample of 5 mice and reported as the mean ± S.E.M. The statistical analysis employed a one-way ANOVA followed by Tukey’s post-test. ^***^ *p* < 0.001 compared to the control group and ^&&^ *p* < 0.01 and ^&&&^ *p* < 0.001 compared to the saline-treated MS group.

The study’s findings revealed that the MS group had a significantly higher number of buried marbles than the control group (*p* < 0.001). However, the number of marbles buried was decreased considerably in the MS mice treated with UMB at a dose of 12.5 mg/kg (*p* < 0.01) and UMB at a dose of 25 mg/kg (*p* < 0.001) compared to the saline-treated MS mice (Figure 2).

### Effects of UMB on sociability and social preference indexes in the three-chamber test

**FIGURE 3:**
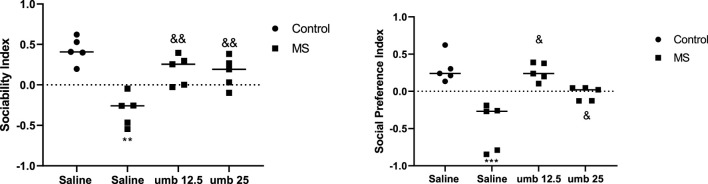
The effect of UMB on sociability index and social preference index. The values were calculated for a sample of 5 mice and reported as the mean ± S.E.M. The statistical analysis employed a one-way ANOVA followed by Tukey’s post-test. ^**^ *p* < 0.01 and ^***^ *p* < 0.001 compared to the control group and ^&^ *p* < 0.05 and ^&&^ *p* < 0.01 compared to the saline-treated MS group.

Findings showed that the MS group exhibited a significant reduction in their SI (*p* < 0.01) compared to the control group. However, when MS groups were treated with UMB at doses of 12.5 and 25 mg/kg, their SI improved significantly (*p* < 0.01) compared to the saline-received MS mice (Figure 3). Additionally, we observed that SPI was decreased considerably in the MS group compared to the control group (*p* < 0.001). When MS groups were treated with UMB at doses of 12.5 and 25 mg/kg, their SPI significantly increased compared to the saline-treated MS animals (*p* < 0.05).

### Effects of UMB on the open arms entries and time in the EPM test 

**FIGURE 4:**
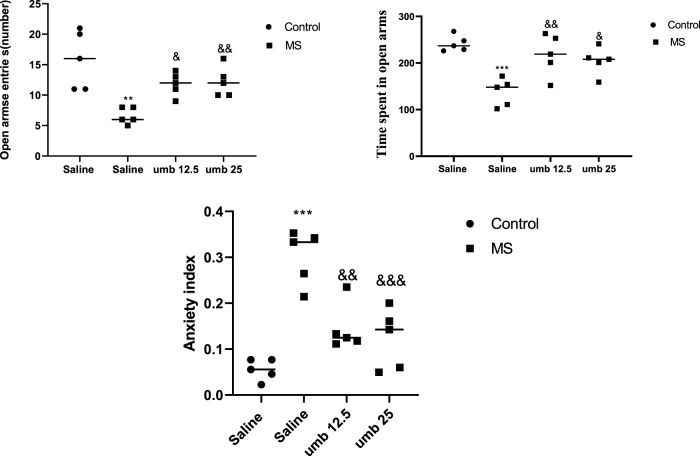
The effect of UMB on the open arms entries and time spent in open arms in the EPM. Values were calculated for a sample of 5 mice and reported as the mean ± S.E.M. The statistical analysis employed a one-way ANOVA followed by Tukey’s post-test. ^**^ *p* < 0.01 and ^***^ *p* < 0.001 in comparison to the control group, ^&^ *p* < 0.05, ^&&^ *p* < 0.01 and ^&&&^ *p* < 0.001 in comparison to the saline-treated MS group.

The findings from the EPM test are presented in Figure 4. The MS group demonstrated a pronounced reduction in open-arm entries compared to the control group (*p* < 0.01). Treatment of the MS group with UMB at doses of 12.5 mg/kg (*p* < 0.05) and 25 mg/kg (*p* < 0.01) resulted in a significant increase in the number of open-arm entries compared to the saline-treated MS mice. Additionally, the time spent in open arms was significantly lower in the MS group compared to the control group (*p* < 0.001). However, administering UMB at a dose of 12.5 mg/kg (*p* < 0.01) and 25 mg/kg (*p* < 0.05) to the MS group resulted in a significant increase in the time spent in open arms compared to the saline-treated MS mice. Furthermore, results showed that the MS group had a higher anxiety index compared to the control mice (*p* < 0.001). Treatment of MS mice with UMB at doses of 12.5 (*p* < 0.01) and 25 mg/kg (*p* < 0.001) significantly decreased anxiety index compared to the saline-treated MS mice.

### Effects of UMB on nitrite levels in the hippocampus

**FIGURE 5:**
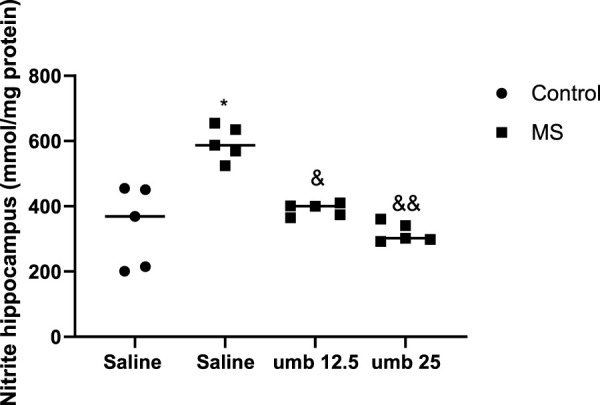
The effect of UMB on the nitrite levels in the hippocampus. Values were calculated for a sample of 5 mice and reported as the mean ± S.E.M. The statistical analysis employed a one-way ANOVA followed by Tukey’s post-test. ^*^ *p* < 0.05 compared to the control group, ^&^ *p* < 0.05 and ^&&^ *p* < 0.01 in compared to the saline-treated MS group.

The results indicated a significant increase in nitrite levels in the hippocampus tissue of the MS group compared to the control group (*p* < 0.05). Treatment of MS mice with UMB at a dose of 12.5 mg/kg (*p* < 0.05) and UMB at a dose of 25 mg/kg (*p* < 0.01) resulted in a significant decrease in nitrite levels compared to the saline-treated MS mice (Figure 5).

### Effects of UMB on MDA levels in the hippocampus

**FIGURE 6:**
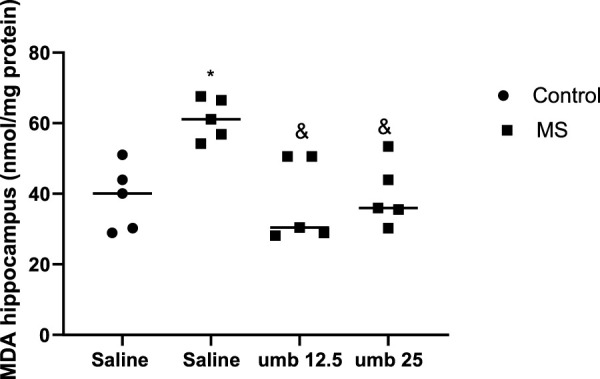
The effect of UMB on the MDA level in the hippocampus. Values were calculated for a sample of 5 mice, reported as the mean ± S.E.M., and analyzed by one-way ANOVA followed by Tukey’s *post hoc* test. ^*^ *p* < 0.05 compared to the control group and ^&^ *p* < 0.05 in compared to the saline-treated MS group.

Based on the results shown in Figure 6, the MDA level of the hippocampus was significantly increased in the MS group compared to the control group (*p* < 0.05). Treatment of MS mice with UMB at doses of 12.5 and 25 mg/kg (*p* < 0.05) resulted in a significant decrease in the MDA levels compared to the saline-treated MS mice.

### Effects of UMB

**FIGURE 7:**
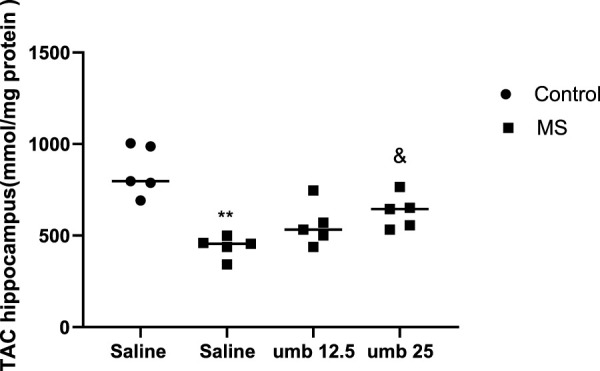
The effect of UMB on the *TAC* in the hippocampus. Values were calculated for a sample of 5 mice, reported as the mean ± S.E.M., and analyzed by one-way ANOVA followed by Tukey’s *post hoc* test. ^**^ *p* < 0.01 compared to the control group and ^&^ *p* < 0.05 in compared to the saline-treated MS group.

on TAC in the hippocampus Results showed that the TAC in the hippocampus of the MS group significantly decreased compared to the control group (*p* < 0.01) (Figure 7). Treatment of MS mice with UMB at a dose of 25 mg/kg resulted in a significant increase in the TAC compared to the saline-treated MS mice (*p* < 0.05).

**FIGURE 8:**
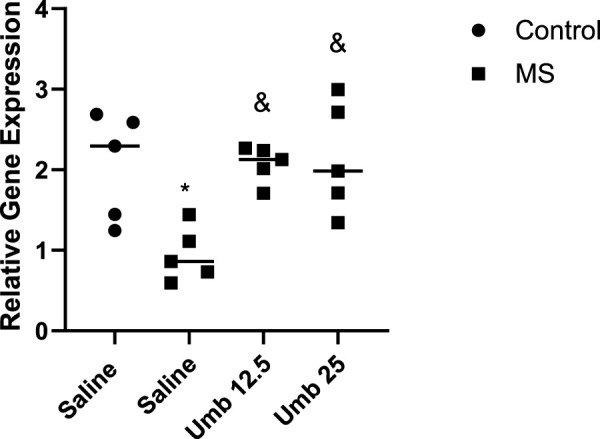
The effect of UMB on the gene expression of *MECP2* in the hippocampus. Values were calculated for a sample of 5 mice, reported as the mean ± S.E.M., and analyzed by one-way ANOVA followed by Tukey’s *post hoc* test. ^*^ *p* < 0.05 compared to the control group and ^&^ *p* < 0.05 in compared to the saline-treated MS group.

Gene expression of *MECP2* in the hippocampus following administration of UMB Figure 8 shows the effects of UMB on the gene expression of *MECP2* in the hippocampus. The results showed that the gene expression of *MECP2* in the hippocampus of the MS group significantly decreased compared to the control group (*p* < 0.05). Treatment of MS mice with UMB at doses of 12.5 and 25 mg/kg resulted in a significant increase in the gene expression of *MECP2* in the hippocampus compared to the saline-treated MS mice (*p* < 0.05).

## Discussion

The present study examined the effects of UMB on the manifestations of autistic-like behaviors following the MS paradigm. Our findings revealed that MS resulted in autistic-like behavior, as evidenced by a decrease in secondary delay time in the shuttle box test, indicating impaired passive avoidance memory. There was also an increase in the number of hidden marbles in the MBT, demonstrating repetitive behaviors. Additionally, the three-chamber test’s sociability and social preference indexes were reduced, suggesting impaired social interaction. Furthermore, there was a decrease in the number and duration of entries in the EPM, reflecting anxiety-like behaviors.

Moreover, we found that autistic-like behaviors are associated with decreased MECP2 gene expression, TAC, and increased MDA and nitrite levels in the hippocampus, indicating an oxidative stress state and neurodevelopmental failure.

Investigating the potential effects of UMB, we observed that UMB significantly attenuated the autistic-like behaviors induced by MS. Furthermore, it increased *MECP2* gene expression. It mitigated oxidative stress markers in the hippocampus.

ASD encompasses a range of neurodevelopmental alterations, and its prevalence is significantly rising worldwide (Selamet and Usta, 2023). Recent studies have elucidated the involvement of multiple environmental and genetic factors in the pathogenesis of ASD (Lipkin et al., 2023). Early detection of Autism can facilitate timely interventions and treatments, improving outcomes (Juruena et al., 2020). The lack of effective treatments, alongside the fact that current therapeutic approaches exhibit partial efficacy and present notable side effects, creates a pressing need to investigate new effective and safe agents for the management of Autism (Vivanti et al., 2014).

Given the incomplete understanding of the pathophysiology of Autism, researchers are actively exploring potential pathways and mechanisms involved in this complex disorder. One pathway of interest is the *MECP2* pathway. Consequently, in this study, we investigated the therapeutic potential of UMB, aiming to elucidate its effect on autistic-like behaviors following the MS paradigm.

Recent investigations have unveiled the profound influence of early-life stress on developing neurodevelopmental abnormalities (Farzan et al., 2023). Furthermore, mounting evidence suggests that early-life stress can contribute to the manifestation of psychiatric disorders and behavioral impairments, including depression, anxiety, and Autism (Amiri et al., 2016; Lorigooini et al., 2020b; Pitsillou et al., 2020). Račekov et al. reported reduced neurogenesis across various brain regions in animals subjected to early postnatal stress (Račeková et al., 2009). In this regard, it has been determined that Autism is associated with decreased neurogenesis in the brain (Bicker et al., 2021). There is growing evidence that ELS, such as MS, is related to the development of ASD (Farzan et al., 2023; Khaledi et al., 2023; Makris et al., 2023). In this regard, it has been demonstrated that MS in animal models induces autistic-like behaviors such as repetitive behaviors, anxiety-like behaviors, memory impairment, and social interaction impairments (Mansouri et al., 2020). Chang et al. revealed an increase in the number of buried marbles in the MBT in mice exhibiting autistic-like features (Chang et al., 2017). In line with the studies mentioned earlier, we showed that maternally separated mice buried more marbles than the control group.

Furthermore, Varadinova et al. observed that in autistic mice, secondary delay in the shuttle box test decreased compared to the control group (Varadinova et al., 2019). Our result showed that maternally separated mice have a lower secondary delay time in the shuttle box than the control mice. Previous studies have reported a decrease in the number and duration of entries in the open arms of EPM for MS mice (Tallarico et al., 2023). Consistent with these findings, our study revealed that MS mice exhibited reduced time and number of entries into the open arms of the EPM. Previous studies have shown that mice with autistic-like features display diminished social interactions, such as lower sociality and social preference indexes, suggesting impaired social communication (Yin et al., 2023). In line with these findings, our study revealed that mice subjected to MS exhibited reduced social communications and connected less with their counterparts compared to the control group. Emerging evidence suggests a role of oxidative stress in the pathophysiology of various neurodevelopmental disorders like ASD (Cipolla and Lodhi, 2017) with particular relevance to ASD (Nadeem et al., 2019). Oxidative stress plays a crucial role in triggering neuroinflammation, which is considered a major contributing factor to ASD (McDougle and Carlezon, 2013). Consistent with these studies, our study revealed a significant decrease in brain antioxidant capacity, as well as an increase in nitrite and MDA levels in the hippocampus.

*MECP2* alteration has been involved in a range of neurodevelopmental disorders like ASD (Wen et al., 2017; Alexander-Howden et al., 2023; Li et al., 2023). Notably, *MECP2* mutations have been extensively described in Rett syndrome, Autism, intellectual disability, and early-onset psychosis (Couvert et al., 2001; Chahrour and Zoghbi, 2007). Furthermore, functional protein alterations resulting from mutated *MECP2* have been linked to distinct neurodevelopmental impairments in Rett syndrome and ASD, which are connected with autistic characteristics (Shahbazian et al., 2002; Shibayama et al., 2004). Our findings align with these reports, demonstrating a significant reduction in *MECP2* gene expression in the hippocampus of MS mice. One limitation of our study is that we only evaluated *MECP2* at the gene level. Assessing *MECP2* at the protein level using Western blotting*, IHC, or ELISA* is suggested for future studies. Another limitation of this study is that we did not examine the effects of umbelliprenin in autistic-like behaviors following MS in female mice.

UMB, a member of the coumarin family, has been determined to exert neuroprotective effects through its antioxidative properties (Sharifi et al., 2020; Fiorito et al., 2022). Recent studies have elucidated its anti-inflammatory, anticancer, immune-modulatory, analgesic, and neuroprotective attributes (Hashemzaei et al., 2015; Rashidi et al., 2018). Ample evidence has corroborated its antioxidative and anti-inflammatory effects (Shakeri et al., 2014).

In the present study, we found that the administration of UMB to the MS mice led to the attenuation of autistic-like behaviors, as indicated by an increase in the social preference index and sociability index in the three-chamber test, an increase in the time and number of entrances to the open arm in the EPM test, an increase in the second delay in the shuttle box test, and a decrease in the number of buried marbles in the MBT. These behavioral tests showed that, at least partially, UMB mitigated autistic-like behaviors following MS. Furthermore, we found that following the administration of UMB, the levels of nitrite and MDA significantly decreased in the hippocampus of the MS mice. In addition, UMB significantly increased total antioxidant capacity in the hippocampus of MS mice. In the case of *MECP2* gene expression, our results showed that UMB significantly increased the gene expression of *MECP2* in the hippocampus of MS mice. However, further studies are warranted to evaluate the exact mechanism underlying the effects of UMB in autistic-like behaviors.

## Conclusion

In conclusion, our results suggest that an increase in the oxidative stress markers and a decrease in the gene expression of *MECP2* in the hippocampus contributes, at least partly, to the manifestation of autistic-like behaviors observed following the MS paradigm. We concluded that UMB probably, partially at least, via attenuation of oxidative stress and increase in the gene expression of *MECP2* in the hippocampus attenuated the autistic-like behaviors following MS stress in male mice.

## Data Availability

The original contributions presented in the study are included in the article/Supplementary Material, further inquiries can be directed to the corresponding author.
